# Emerging role of Garcinol, the antioxidant chalcone from *Garcinia indica *Choisy and its synthetic analogs

**DOI:** 10.1186/1756-8722-2-38

**Published:** 2009-09-02

**Authors:** Subhash Padhye, Aamir Ahmad, Nikhil Oswal, Fazlul H Sarkar

**Affiliations:** 1 Department of Pathology, Barbara Ann Karmanos Cancer Center and Wayne State University School of Medicine, Detroit, MI 48201, USA; 2 D.Y. Patil University of Pharmaceutical Sciences and Research, Pune 411018, India; 3 D.Y. Patil Institute of Pharmaceutical Sciences and Research, Pune 411018, India

## Abstract

Garcinol, harvested from *Garcinia indica*, has traditionally been used in tropical regions and appreciated for centuries; however its biological properties are only beginning to be elucidated. There is ample data to suggest potent antioxidant properties of this compound which have been used to explain most of its observed biological activities. However, emerging evidence suggests that garcinol could be useful as an anti-cancer agent, and it is increasingly being realized that garcinol is a pleiotropic agent capable of modulating key regulatory cell signaling pathways. Here we have summarized the progress of our current research knowledge on garcinol and its observed biological activities. We have also provided an explanation of observed properties based on its chemical structure and provided an insight into the structure and properties of chalcones, the precursors of garcinol. The available data is promising but more detailed investigations into the various properties of this compound, particularly its anti-cancer activity are urgently needed, and it is our hope that this review will stimulate further research for elucidating and appreciating the value of this nature's wonder agent.

## Introduction

It is difficult to imagine that the pink sweet smelling drink that is served to the world travelers spending summer holidays on the beautiful beaches of Goa in India, upon their arrival at the hotel, could one day end up on the laboratory tables of Cancer Institutes around the world. The welcome drink happens to be made from the syrup formulated from the fruits locally known as 'Kokum' which is steeped in sugar syrup to make a drink which is used to avoid skin damages and allergies from the sun and tropical climate. The plant grows extensively on the western coast of India and is known by various names across India including Bindin, Biran, Bhirand, Bhinda, Katambi, Panarpuli, Ratamba or Amsool. In English language, it is also known by various names such as Mangosteen, wild Mangosteen, or Red Mango. According to botanical classification the tree is classified as *Garcinia indica *(Family: Clusiaceae; Genus: Garcinia)which has many culinary, pharmaceutical and industrial uses. The genus Garcinia includes some 200 species found in the tropics, especially Asia and Africa. Out of 35 species found in India, 17 are endemic. Of these, seven are endemic to the region of Western Ghats including the state of Goa, six in the Andaman and Nicobar Islands and four in the North-Eastern region of India.

The *Garcinia indica *seed contains 23-26% oil, which remains solid at room temperature and is used in the preparation of chocolates, medicines and cosmetics. It is used as a slightly bitter spice in recipes from the state of Maharashtra in India and as a souring agent and a substitute for tamarind paste in Indian curries. Recently, some industries have started extracting hydroxycitric acid (HCA) from the rind of the fruit which is an important constituent used as a hypocholesterolaemic agent. HCA is also a potential anti-obesity agent [1]. It suppresses fatty acid synthesis, lipogenesis, and food intake, and thus induces weight loss. Kokum Butter is an excellent emollient used by the cosmetic industry for preparations of lotions, creams, lip-balms and soaps. It has relatively high melting point and is considered as one of the most stable exotic butter which dose not need any refrigeration. It is extracted from the Kokum seed and is supposed to reduce degeneration of the skin cells and restore elasticity. The extract of the plant finds place in the specialty cuisine of West Coast of India as an appetizer while decoction of the bark is used for treating paralysis. The antioxidant activity of aqueous extract of the plant has been reported, which is higher than other reported spices and fruits thus promoting its use in cooking, home remedies and as a soft drink [2]. *Garcinia indica *extract has also been shown to inhibit *Aspergillus flavus *and aflatoxin B_1 _production thereby demonstrating its putative bio-preservative properties [3]. Addition of Garcinia extract to fresh skipjack (dark muscle fish) has been demonstrated to prevent histamine formation by lowering the pH to 3.2-3.6 [4]. Since histamine is known to give rise to allergic reactions, Garcinia extracts can potentially find use in anti-allergy medications.

## Isolation and characterization of chemical constituents

**Figure 1:**
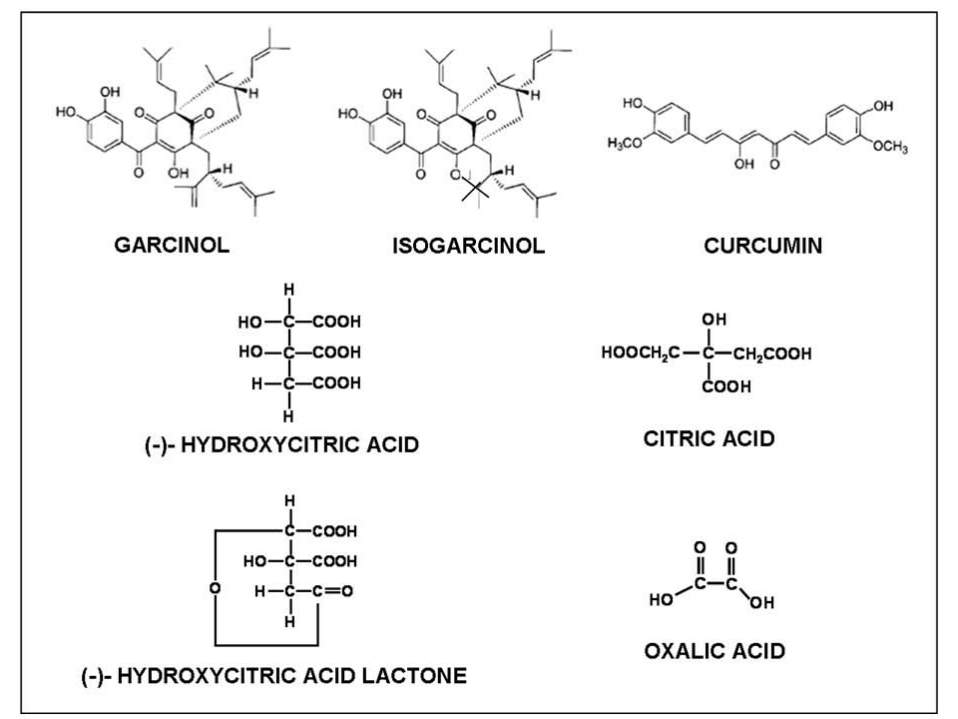
**Structure of Garcinol, Curcumin and compounds extracted from *Garcinia indica***.

*Garcinia indica *extracts, especially from its rind, are rich in polyisoprenylated benzophenone derivatives such as Garcinol and its colorless isomer, Isogarcinol. The rind also contains hydroxycitric acid (HCA), hydroxycitric acid lactone, citric acid and oxalic acid. The structures of these compounds are shown in Figure 1. The fruit also contains other compounds including malic acid, polyphenols, carbohydrates, anthocyanin, pigments and ascorbic acid. Garcinol shows strong antioxidant activity since it contains both phenolic hydroxyl groups as well as a β-diketone moiety, and in this respect it resembles with the well-known antioxidant of plant origin, viz. Curcumin [5] (Figure 1).

A reverse-phase high-performance liquid chromatographic method has been developed by Chattopadhyay and Kumar for qualitative and quantitative analysis of Xanthochymol and Isoxanthochymol in the fruit rinds, leaves and seed pericarps of *Garcinia indica *using PDA detector and electrospray ionization mass spectra. Absorption at 276 nm was chosen as the measuring wavelength at which resolution of both compounds could be obtained [6-9]. More recently, these workers have developed a rapid, sensitive and simple reverse-phase high-performance liquid chromatography-electrospray ionization mass spectrometric method for the identification and quantification of two isomeric polyisoprenylated benzophenones, isoxanthochymol and camboginol, in the extracts of the stem bark, seeds and seed pericarps of *Garcinia indica *and in the fruit rinds of *Garcinia cambogia *[10]. The major organic acid in leaves and rinds of *Garcinia indica *is reported to be (-)-hydroxycitric acid, present to the extent of 4.1-4.6 and 10.3-12.7% respectively, as determined by HPLC [11,12].

Garcinol, with a molecular weight of 602, is the active principle of *Garcinia indica*, which is crystallized out as yellow needles (1.5%) from the hexane extract of the fruit rind. The molecular formula and the absorption spectral data indicate that the compound is possibly related to the isomeric Xanthochymol and more appropriately, in view of the sign of optical rotation, to Cambogin. The presence of an enolisable 1, 3-diketone system in the molecule is confirmed by the formation of two isomeric trimethyl ethers, hydrolysable to single dimethyl ether with dilute alkali. Alkali degradation of the methyl ether under stronger conditions (20% ethanolic KOH, reflux) yields veratric acid indicating the presence of a 3,4-dihydroxybenzoyl unit. The UV spectrum of garcinol suggests that the 1, 3-diketone system is conjugated to the 3, 4-dihydroxybenzoyl moiety. The IR spectrum of the trimethyl ether shows the presence of a saturated carbonyl group (1727 cm^-1^) and two α, β-unsaturated carbonyl groups (1668 and 1642 cm^-1^), accounting for all the oxygen atoms.

The PMR spectrum of garcinol in CDC1_3 _shows the presence of two saturated tertiary methyls (singlets at δ l.01 and 1.17) and seven = C-CH_3 _groups (signals at δ 1.54 for two methyls and at 1.60, 1.67, 1.70, 1.74 and 1.84 for one methyl each). It also shows signals for a vinylic methylene (δ 4.38, 2 H, broad singlet) and three other olefinic protons (δ 5.0 m) in addition to three aromatic protons (ABX pattern around δ 6.60 and 6.95) and a hydrogen bonded phenolic hydroxyl at δ 18.0. The mass spectrum of garcinol is very similar to that of Xanthochymol exhibiting major peaks at m/e 602(M^+^), 465(M^+ ^-C_10_H_17_, base peak), 341 (465-C_9_H_16_) and 137 (Dihydroxybenzoyl). These features clearly indicate that the structure of garcinol is biogenetically derivable from Maclurin (2,4,6,3',4'-pentahydroxybenzophenone) and five isoprenyl units [13,14].

## Chemistry of garcinol

The principle antioxidant substance of *Garcinia indica *and other species is Garcinol (Figure 1) also called as Camboginol, which is a tri-isoprenylated chalcone [15,16]. This compound is extracted from the dried fruit rind of the plant. It scavenges 1, 1-diphenyl-2-picrylhydrazyl (DPPH) free radical (3 times more effectively than DL-R-tocopherol), hydroxyl radical (more effectively than DL-R-tocopherol), methyl radical, and superoxide anion [17]. Sang *et al*. have reported the structure of some oxidation products of garcinol and have proposed mechanisms for the formation of these products [18,19]. Their results suggest that garcinol can play an important role in the treatment of gastric ulcers caused by the hydroxyl radicals or chronic infection with *Helicobacter pylori*, which, together with cells from gastric mucous membrane, produces hydroxyl radicals and superoxide anions. Presently, treatment with Clarithromycin antibiotic is the therapy of choice for treating *H. pylori *infection, which, however, suffers from side effects and emergence of rapid resistance [20,21]. Garcinol may be a viable alternative to conventional antibiotics. Garcinol shows antibacterial activity against Methicillin-resistant *Staphylococcus aureus *[22] which is comparable to that of the antibiotic Vancomycin (MIC - 3-12 *μ*g/mL for garcinol Vs. 6 *μ*g/mL for Vancomycin) [23]. It also inhibits topoisomerases I and II (IC_50 _= 43 and 55 *μ*g/mL respectively) at concentrations comparable to that of Etoposide (IC_50 _= 70 *μ*g/mL for topoisomerases II) [24]. Although this compound has been shown to exhibit therapeutic activity against gram-positive and gram-negative cocci, mycobacteria and fungi, it has been found to be inactive against gram-negative enteric bacilli, yeasts and viruses [25]. Garcinol exerts anti-cholinesterase properties towards acetyl cholinesterase (AChE) and butylcholinesterase. The IC_50 _value of garcinol (0.66 μM) against AChE is comparable to that of the reference compound Galanthamine (0.50 μM) [26].

Isogarcinol also shows biological activities similar to that of garcinol and has been claimed to be an anti-inflammatory and antitumor compound, a lipase inhibitor, an anti-obesity agent as well as an antiulcer agent [18]. Sang *et al. *have studied the interaction of garcinol with peroxyl radicals generated by thermolysis of the initiator 2, 2'-azobis-isobutyronitrile (AIBN) and have succeeded in isolating and characterizing reaction products of garcinol in a homogeneous acetone system. The resulting compounds were found capable of inducing apoptosis in human leukemia HL-60 cells and inhibit NO radical generation as well as LPS-induced iNOS gene expression, respectively [18,19]. Garcinol showed good antitumor activity against human leukemia HL-60 cells, being more effective than curcumin, which was used as a reference compounds in these studies. In addition to HL-60 cells, the chemotherapeutic potential of garcinol has been examined on other cell lines as well such as murine macrophage RAW 264.7 cells and cyclin D1-positive cells showing similar results. Additionally garcinol also inhibits histone acetyltransferases (HATs, IC_50 _= 7 *μ*M) and p300/CPB-associated factor (PCAF, IC_50 _= 5 *μ*M), both of which are known to modulate gene expression [27].

## Biological activities of garcinol

### a. Antioxidant Activity

Garcinol has been shown to possess antioxidant activity in the H_2_O_2_-NaOH-DMSO system as well as the radical scavenging activity against superoxide anion, hydroxyl radical and methyl radical respectively. The emulsified garcinol suppresses superoxide anion to almost same extent as DL-α tocopherol by weight, while it exhibits nearly three times greater free radical scavenging activity against 2, 2, diphenyl-1-picrylhydrazyl (DPPH) radicals than DL-α tocopherol by weight [28]. The following paragraphs describe the known mechanism of antioxidant activity of garcinol.

Hong *et al. *have investigated possible mechanisms of antioxidant action of garcinol and its derivatives on arachidonic acid metabolism and NO radical synthesis at concentrations (>1 μM) that may be achievable under in *vivo *conditions. The preliminary results indicate that peak plasma and urine plasma concentration levels of garcinol in CD-1 female mice were 12 and 2.7 *μ*M respectively, after oral gavage of garcinol (10 mg dose per mouse) [29]. Sang *et al. *also proposed the antioxidant mechanism of garcinol according to which the compound reacts with peroxyl radicals by a single electron transfer followed by deprotonation of the hydroxyl group from the enolized 1, 3-diketone to form a resonance pair. Depending on the position of hydroxyl group (C-3 or C-5) which initiates the reaction, different compounds are formed [18,19].

The neuroprotective effects of garcinol were examined by Liao *et al *who found that at 5 μM concentration it prevented NO radical accumulation in LPS-treated astrocytes and significantly reduced the expression of LPS-induced inflammatory mediators, such as iNOS and COX-2 [30]. These results suggest that the neuroprotective effects of garcinol are associated with its antioxidant nature involving inhibition of iNOS induction in astrocytes. It has been suggested that the compound may be neuroprotective against brain injury through similar mechanism [30]. Yamaguchi *et al. *studied various pharmacological activities of garcinol including antioxidant activity, chelating activity, free radical scavenging activity and anti-glycation activity. They observed that garcinol exhibited reasonable antioxidant activity in the micellar linoleic acid peroxidation system and exhibited chelating activity at almost the same level as citrates. In a phenazine methosulfate/NADH-nitro blue tetrazolium system garcinol exhibited superoxide anion scavenging activity and suppressed protein glycation in a bovine serum albumin/fructose system. Thus, the compound may be useful as a glycation inhibitor under specified conditions [17].

### b. Anti-inflammatory activity

Aberrant arachidonic acid metabolism and generation of nitric oxide radicals (NO) have been shown to be involved in inflammation and carcinogenesis [29]. Arachidonic acid is released by phospholipase A_2 _(cPLA2) from membrane phospholipids and is further metabolized by cyclooxygenase (COX), lipooxygenase (LOX) enzymes and Cytochrome P450 pathways. Modulation of arachidonic acid metabolism by inhibiting COX and LOX enzymes has been considered as an effective approach for treating inflammation and for cancer chemoprevention [29]. Garcinol and its derivatives modulate arachidonic acid metabolism by retarding the phosphorylation of cytosolic PLA2 (cPLA_2_) through the inhibition of extracellular ERK1/2 kinase activation and suppression of iNOS expression through modulation of the Janus kinase (JAK)/STAT-1 signaling pathway. When added prior to LPS, garcinol suppressed NF-κB activation and COX-2 expression through the interruption of LPS binding to toll-like receptors [29].

The nitric oxide radical moiety is involved in various physiological processes, including vasodilation, inhibition of platelet function, synaptic neurotransmission as well as host defense. The formation of NO radicals from arginine in the biological system is catalyzed by three different types of nitric oxide synthase (NOS) enzymes, viz. endothelial NOS, neuronal NOS and inducible NOS (iNOS), respectively. The enzyme iNOS is stimulated by inflammatory cytokines for NO production by macrophages and by many other cell types. It has been reported that garcinol inhibits the expression of iNOS and COX-2 in lipopolysaccharide (LPS)-activated macrophages [31]. It was observed that garcinol strongly blocks the LPS-induced activation of eukaryotic transcription factor NF-κB [31]. This inhibition of NF-κB activation takes place through the suppression of phosphorylation of IκB-α and p38 Mitogen-Activated Protein Kinases (MAPK). Garcinol lowers the LPS-induced increase of intracellular reactive oxygen species (ROS), which contribute to the activation of NF-κB [31].

Recently Koeberle *et al. *have shown that garcinol significantly interferes with two enzymes that play crucial roles in inflammation and tumorigenesis, viz. 5-lipoxygenase and microsomal prostaglandin PGE_2 _synthase (mPGES)-1 [32]. In cell-free assays garcinol inhibits the activity of purified 5-lipoxygenase and blocks the mPGES-1-mediated conversion of PGH_2 _to PGE_2 _with IC_50 _values of 0.1 and 0.3 μM respectively. Garcinol was found to suppress 5-lipoxygenase product formations in intact human neutrophils and reduced PGE_2 _formation in interleukin-1β-stimulated A549 human lung carcinoma cells as well as in human whole blood stimulated by lipopolysaccharide. Garcinol also interfered with isolated COX-1 enzyme (IC_50 _= 12 μM) and with the formation of COX-1-derived 12(*S*)-hydroxy-5-cis-8, 10-trans-heptadecatrienoic acid as well as thromboxane B_2 _in human platelets. The high potency of garcinol in selectively suppressing PGE_2 _synthesis and 5-lipoxygenase product formations provides the molecular basis for its anti-inflammatory and anti-carcinogenic effects and rationalizes its therapeutic use [32].

### c. Anticancer activity

The effects of garcinol and its oxidative derivatives have been investigated on the growth of HT-29 and HCT-116 colon cancer cells, as well as IEC-6 and INT-407 which are the normal immortalized intestinal cells [33]. Garcinol and its derivatives showed potent growth-inhibitory effects on all intestinal cells, with IC_50 _values in the range of 3.2-21.4 μM after 72 hr treatment. Garcinol was found to be more effective in inhibiting growth of cancer cells than that of normal immortalized cells. These results indicate that garcinol and its derivatives can inhibit intestinal cancer cell growth without affecting normal cells. However, it should be pointed out that at low concentrations garcinol can stimulate cell growth [33]. An earlier study investigated the modifying effects of dietary feeding of the compound on the development of azoxymethane (AOM)-induced colonic aberrant crypt foci (ACF) in male F344 rats [34]. The study also assessed the effects of garcinol on proliferating cell nuclear antigen (PCNA) index in ACF and activities of detoxifying enzymes such as glutathione S-transferase (GST) and quinone reductase (QR) in liver. It was observed that garcinol administration significantly lowers PCNA index in ACF and significantly elevates liver GST and QR activities. In addition, garcinol was also found to suppress O(2)(-) and NO generation and expression of iNOS and COX-2 proteins. These observations suggest possible chemopreventive role of garcinol [34]. In yet another report on the suppression of ACF formation in rats by garcinol [35], the beneficial effects of garcinol against tumor prevention in human colorectal cancer cell line, HT-29 were investigated. Matrigel analysis showed that exposure of HT-29 cells to 10 μM garcinol inhibited cell invasion and decreased the dose-dependent tyrosine phosphorylation of focal adhesion kinase (FAK). Western blot analysis demonstrated that garcinol inhibits activation of the Src, MAPK/ERK, and PI3K/Akt signaling pathways. Additionally, these studies demonstrated that decreased MMP-7 protein levels in HT-29 cells result in sensitization to garcinol and that the compound significantly inhibits the expression of MMP-7 in IL-1beta-induced HT-29 cells. Thus, garcinol reduces cell invasion and survival through the inhibition of FAK's downstream signaling [35].

In human leukemia HL-60 cells, garcinol has been reported to display strong growth inhibitory activity (IC_50 _= 9.42 μM) through induction of caspase-3/CPP32 activity in a dose- and time-dependent manner and inducing degradation of poly (ADP-ribose) polymerase (PARP) protein [5]. This induction of apoptosis provides a pivotal mechanism for its cancer chemopreventive action. In a study comprising four human leukemia cells lines, loss of mitochondrial membrane potential was observed during garcinol-induced apoptosis [36]. Garcinol also modulates arachidonic acid metabolism by blocking the phosphorylation of cPLA2 and by decreasing iNOS protein level mediated via inhibition of STAT-1 activation. These activities may contribute to the anti-inflammatory and anti-cancer properties of garcinol and its derivatives [29].

Two new benzophenones corresponding to the 13-O-methyl ethers of garcinol and isogarcinol were tested for their inhibitory effects on Epstein-Barr virus early antigen activation induced by 12-O-tetradecanoylphorbol-13-acetate (TPA) in Raji cells and their radical-scavenging ability against 1,1-diphenyl-2-picrylhydrazyl (DPPH) was demonstrated [37]. The cyclized polyprenylbenzophenones showed comparable or stronger potential cancer chemopreventive activity when compared to glycyrrhetic acid, a known anti-tumor promoter. Yoshida and coworkers have demonstrated that dietary garcinol significantly decreases the incidence and multiplicity of 4-NQO-induced tongue neoplasms and pre-neoplasms as compared to the control diet [38]. It also significantly reduced the BrdU-labeling index and cyclin D1-positive cell ratio, suggesting reduction in cell proliferation activity in the tongue. The COX-2 expression in the tongue lesions was also suppressed. They concluded that dietary administration of garcinol inhibits 4-NQO-induced tongue carcinogenesis through suppression of increased cell proliferation activity in the target tissues and/or COX-2 expression in the tongue lesions [38].

The potent cytotoxic activity for the methanol extract of the fruit rinds of *Garcinia indica *against three human cancer cell lines, viz. colon (COLO-320-DM), breast (MCF-7) and liver (WRL-68) has been reported [39]. Fractionation of the methanol extract into hexane-, chloroform- and ethyl acetate-soluble portions was performed and their cytotoxic activity was evaluated. The ethyl acetate fraction was found to be the most effective as compared to the two other fractions. Thus, current results provide evidence for the potential of garcinol as a chemopreventive agent in carcinogenesis. Additionally, feeding garcinol-containing diets does not cause retardation of body weight gain and pathological alterations in liver and other organs including kidney, lung, heart, and esophagus, which is indicative of the low toxicity of the compound, which is a very attractive feature of any anti-cancer agent.

### d. Anti-HIV Activity

Histone acetylation is a diagnostic feature of transcriptionally active genes [40]. The proper recruitment and function of histone acetyltransferases (HATs) and histone deacetylases (HDACs) are key regulatory steps for gene expression and cell cycle. Functional defects of either of these enzymes may lead to several diseases, including cancer. It has been reported that garcinol is a potent non-specific inhibitor of histone acetyltransferases p300 (IC50 = 7 μM) which strongly inhibited HAT activity-dependent chromatin transcription, whereas transcription from DNA template was not affected [40]. In order to find out more potent, specific, and less toxic inhibitors, Mantelingu *et al*. [41] synthesized and characterized several derivatives of isogarcinol (IG), a product of intramolecular cyclization of garcinol, by controlled modification and mono-substitution at C-14 position. In this way they were able to synthesize 14-isopropoxy IG (LTK-13) and 14-methoxy IG (LTK-14) derivatives of isogarcinol. The di-substitution yielded 13, 14 di-isopropoxy IG (LTK-13A), 13, 14 di-methoxy IG (LTK-14A), 13, 14 di-acetoxy IG (LTK-15) and 13, 14 di-sulfoxy (LTK-19) isogarcinol compounds, respectively. It was found that the mono-substituted isogarcinol derivatives like LTK-13, -14, and di-substituted LTK-19 derivative could inhibit the p300-HAT activity but not the PCAF-HAT activity, although the parent isogarcinol compound inhibited HAT activities of both p300 and PCAF non-specifically.

Interestingly, one of the derivatives, LTK-15, seemed to loose the HAT inhibition activity: it could inhibit the p300-mediated acetylation less than 10% and had no effect on PCAF-HAT activity. Furthermore, the other di-substituted isogarcinol derivatives, LTK-13A and LTK-14A, also lost their activity completely. The IC_50 _values, of LTK-13, -14, and -19, to inhibit p300- HAT activity were found to be 5-7 μM, which is comparable to isogarcinol. In order to visualize the inhibition pattern of histone acetylation, HAT assay products were analyzed by fluorography followed by autoradiography. In agreement with filter-binding data, it was found that in the presence of 10 μM of LTK-13, -14, and -19, the p300-mediated acetylation of histones H3 and H4 were equally inhibited up to 85%-90% as compared to DMSO solvent control. The histone acetylation by PCAF (predominantly at histone H3) was not affected by LTK-13, -14, and -19. As expected, the presence of 10-μM concentration of isogarcinol efficiently inhibited histone acetylation by both p300 and PCAF. However, dose-dependent inhibition of p300- HAT activity was observed in the presence of LTK-14. Significantly, HAT-activity of PCAF remained unchanged even in the presence of 50 μM LTK14 and these chromatin modifying enzyme activities were not affected by the presence of isogarcinol and its derivatives. Taken together, the data suggests that the isogarcinol derivatives are specific inhibitors of p300-HAT activity [41,42].

Since reversible acetylation of histone and non-histone proteins plays pivotal role in cellular homeostasis [43], dysfunction of histone acetyltransferases (HATs) is known to cause several diseases including cancer, neurodegenaration, asthma, diabetes, AIDS, and cardiac hypertrophy. Moreover, since p300 protein plays a critical role in cell growth, differentiation, and death, several of these functions require intrinsic HAT activity of p300-HAT; however, the molecular basis of p300 contribution toward diverse cellular processes is still unresolved [43]. Mantelingu *et al*. [41] have described the synthesis and characterization of a set of p300-HAT-specific small-molecule inhibitors derived from garcinol that are highly toxic to cells. They have shown that these specific inhibitors selectively block the p300-mediated acetylation of p53 *in vivo*. Furthermore, inhibition of p300-HAT down-regulates several genes but, significantly, few important genes are also up regulated. Remarkably, these inhibitors were found to be non-toxic to T cells, while inhibiting histone acetylation of HIV infected cells and consequently inhibiting the multiplication of HIV. Hence, garcinol holds tremendous therapeutic potential for different diseases including AIDS and cancer.

### e. Anti-ulcer activity

Garcinol has potent free radical scavenging activity as judged from its interactions in three types of free radical generating systems. Its scavenging activity against hydroxyl radical has been found to be stronger than that of α-Tocopherol [17] while its other scavenging activities were found to be slightly weaker. Since hydroxyl radical is regarded as the most damaging Reactive Oxygen Species (ROS), garcinol is expected to be useful for preventing diseases caused by the hydroxyl radical damages such as stress-induced gastric ulcer [44,45] and NSAID drug-induced gastric ulcers [46,47]. In the water immersion stress model, Yamaguchi et al. have shown that garcinol suppressed gastric injury formation to almost same extent as cetraxate hydrochloride as a positive control [28]. It also prevented indomethacin-induced gastric injury. These results suggest that garcinol may have potential as an anti-ulcer drug. Although mechanism of its anti-ulcer activity is not yet understood, it may be speculated that the compound may scavenge reactive oxygen species on the surface of gastric mucosa, thus protecting cells from injury [28].

## Structure-activity considerations for garcinol

It has been clearly established that the C-3 kenotic group and the phenolic ring bearing hydroxyl group are the principal oxidation sites of garcinol generating its oxidized products during metabolic transformations some of which are also biologically active [18,19]. It has also been found that the 1, 2 carbon-carbon double bond of the α, β-unsaturated ketone is important for apoptosis-inducing activity and cytotoxicity of garcinol [5]. The double bond of the isoprenyl group is also a principal site of the antioxidant reaction of garcinol; however, compounds without having such substitution and bearing structural resemblance to garcinol, like curcumin, have been found to be potent antioxidants [48]. The isoprenyl chain of garcinol consists of hydrophobic faces, which are important for its binding to biological targets [49].

## Chalcones as a garcinol analoges

Kostanecki, who pioneered work in the synthesis of natural coloring compounds, first coined the term 'chalcone'. An interesting feature of chalcones is that they serve as starting materials for another class of naturally occurring and widely distributed pigments, flavones [50]. They are considered to be precursors of flavonoids and isoflavonoids, which are abundant in edible plants. Chalcones are intermediates in the synthesis of flavones. Chemically they are open-chain flavonoids in which the two aromatic rings are joined by a three-carbon α, β-unsaturated carbonyl system (1, 3-diphenyl-2-propen-1-one). Chalcones exhibit many pharmacological activities including anti-leishmanial [51], anti-inflammatory [52,53], anti-mitotic [54], anti-invasive [55], anti-tuberculosis [56], anti-fungal [57], cysteinyl leukotriene receptor-1 antagonist [58], anti-malarial [59,60], anti-plasmodial, antitumor, immunosuppressive, antioxidant [61], anti-fibrogenic and modulation of P-glycoprotein-mediated multi-drug resistance [62]. Recent studies have shown that chalcones inhibit cancer cell proliferation *in vivo *and are effective agents against skin cancers [63,64]. They also induce apoptosis in various cell types, including breast cancers [65]. Several oxygenated chalcones; hydroxyl chalcones, bis-chalcones and quinolinyl chalcone analogs exhibit anti-malarial activity [66,67]. Some chalcones also demonstrate their ability to block voltage-dependent potassium channels [68]. These limited yet interesting studies clearly suggest the beneficial effects of chalcones and other derivatives in human health and diseases.

### a. Structural chemistry of chalcones

**Figure 2:**
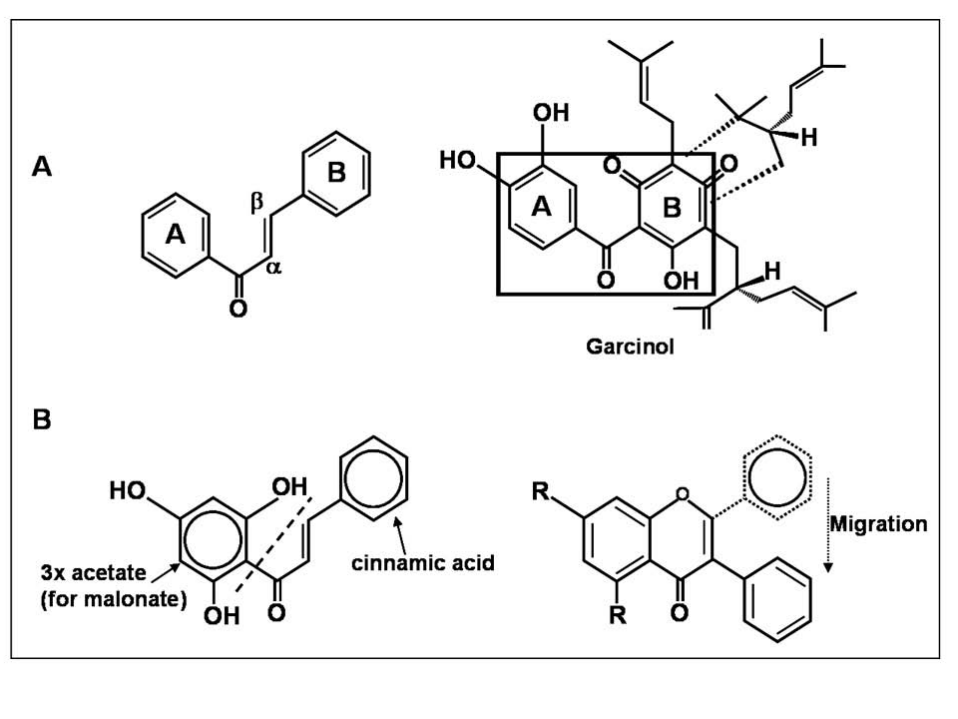
**A. Structural similarity between chalcone and garcinol moieties**. **B**. Formation of chalcone and migration of ring B.

Chalcones consist of two aromatic rings in *trans *configuration, separated by three carbons, of which two are connected by double bond while the third is a carbonyl group [69]. Garcinol is an example of prenylated chalcones, containing two aromatic rings separated by carbonyl group (Figure 2), which is structurally similar to curcumin that resembles chalcones when opened [5]. Genealogical studies have shown that chalcones have evolved prior to garcinol, and chalcones are derived from three acetates and cinnamic acid as shown in Figure 2. Since chalcones are efficient precursors of isoflavonoids, the required aryl migration of ring B from the beta position to the alpha position of the phenylpropanoid precursor must take place after formation of the basic C_15 _skeleton [70]. A vast number of naturally occurring chalcones are polyhydroxylated in the aryl rings. The radical quenching properties of the phenolic groups present in many chalcones have raised interest in using these compounds as therapeutic agents or food preservatives [71].

**Figure 3:**
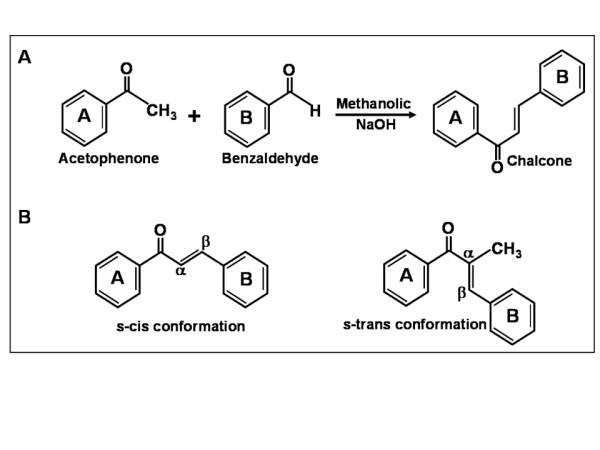
**A. Scheme of synthesis of chalcones**. **B**. s-cis and s-trans conformation of chalcones.

Chalcones are readily synthesized by the base-catalyzed Claisen-Schimdt condensation of an aldehyde and appropriate ketone in a polar solvent like methanol or ethanol (Figure 3) [61]. The synthesis of hydroxylated chalcones by the Claisen-Schimdt method requires protection of the phenolic hydroxyl groups on aldehyde and ketone (except ortho-hydroxyl groups), generally as tetrahydropyranyl (THP), methoxymethyl (MOM), or methoxyethoxymethyl (MEM) ethers. The MOM and MEM ethers are cleaved in the presence of acid, under such conditions; and hence the side reactions compromise the yield of the final product [72]. The Cα-Cβ double bond in the 'enone' moiety of chalcones can adopt Z or E configuration. The E-isomer is thermodynamically more stable and almost all chalcones are isolated in this form. Iwata and co-workers have reported isomerization of E-chalcone to the Z form by exposing the methanolic solution of the chalcone to normal visible light [73]. Interestingly, the Z isomer showed more potent antitumor activity than the original E form. Photoisomerization of the predominant E isomer to the Z isomer may cause change in biological activity and the ease with which the reaction occurs suggest that it is prudent to protect solution of chalcones from light.

Ducki et al. have noted that the two bonds were positioned *cis *with respect to each other in several X-ray crystal structures of chalcones [54]. The s-cis conformer was more stable than the s-trans conformer by, at least, 3.9 kJ/mol. On the other hand, when a methyl group was introduced at the Cα position, the disposition of the carbonyl and Cα-Cβ double bonds altered to the *trans *orientation. For these α-methyl chalcones, molecular mechanics calculations showed that the minimum energy conformers were s-trans and no s-cis conformation was evident within a 10-kJ/mol range of the global energy minimum. The α-methyl group also caused significant loss of planarity between ring A and the enone (θ1 56-88°). The α-methylchalcones are found to have greater cytotoxic activity against a human leukemia cell line than the unsubstantiated analogues. Their unique geometrical features were cited as a possible factor contributing to the enhanced biological activity.

### b. Biological Activities of chalcones

Xia and coworkers were the first to demonstrate improved anti-proliferative activity of chalcones with substituted amino groups [74]. LeBlanc *et al. *have shown that methoxylated chalcones with a 3'-amino group had sub-micromolar IC_50 _values against murine melanoma B16 cells [75]. Dimmock and coworkers proposed that the presence of amino function increases the reactivity of chalcones as Michael acceptors and subsequently their anti-proliferative activity [76]. They postulated that the amino function would be protonated at low pH environment normally encountered in tumors. The electron withdrawing effect of the protonated ammonium function would enhance the electrophilicity of the β-carbon in the enone linkage, hence increasing its reactivity as a Michael acceptor [77]. Liquorice has been used in China for the treatment of gastric and duodenal ulcers, bronchial asthma, Addison's disease, poisoning by food and drugs and skin disease such as eczema and urticaria [78]. It still finds medicinal application because of its wide-ranging therapeutic properties, including relief from rheumatic and other types of pain and healing effect on ulcers. The crude extract of Liquorice has also found commercial use as a food additive in Japan since it contains the sweetening principle glycyrrhizin. The Liquorice extracts contains a chalcone, viz. Isoliquritigenin, which is currently in use as a phosphodiesterase III inhibitor for the treatment of cardiovascular diseases [79]. In the Far East countries such as Korea, Japan, and China, another chalcone compound called 'Butein' has also been traditionally used for treatment of pain, thrombotic disease, stomach cancer, and parasitic infection as well as a food additive [80].

Anti-angiogenic effect of xanthochymol and Isoxanthochymol, the chalcones isolated from the hop, has been reported [81]. A dose-dependant reduction of newly formed capillary growth by xanthochymol was observed, at a concentration range of 0.5-10 μM (IC_50 _value of 2.2 μM) under *in vitro *conditions. Later, it was shown that xanthochymol repressed both the NF-κB and Akt pathways in the endothelial cells, indicating that components of these pathways are major targets in the molecular mechanism of this compound [82]. Xanthochymol also reduced VEGF secretion, decreased cell invasion and metalloprotease production in acute and chronic myelogenous leukemia cell lines [83]. 2'-hydroxychalcones, 4'hydroxychalcones and 2', 4'-dihydroxychalcones inhibit 12-Lipoxygenase and cyclooxygenase enzymes in the mouse epidermis [84] and two synthetic 2'-hydroxychalcones that exert topical anti-inflammatory effects in mice have also been reported [85]. The good selective inhibitory effects of 2', 5' dihydroxychalcones on arachidonic acid-induced platelet aggregation have been suggested [86] and these reports, taken together, suggest that some hydroxy chalcones might be promising antithrombotic or anti-inflammatory agents.

**Figure 4:**
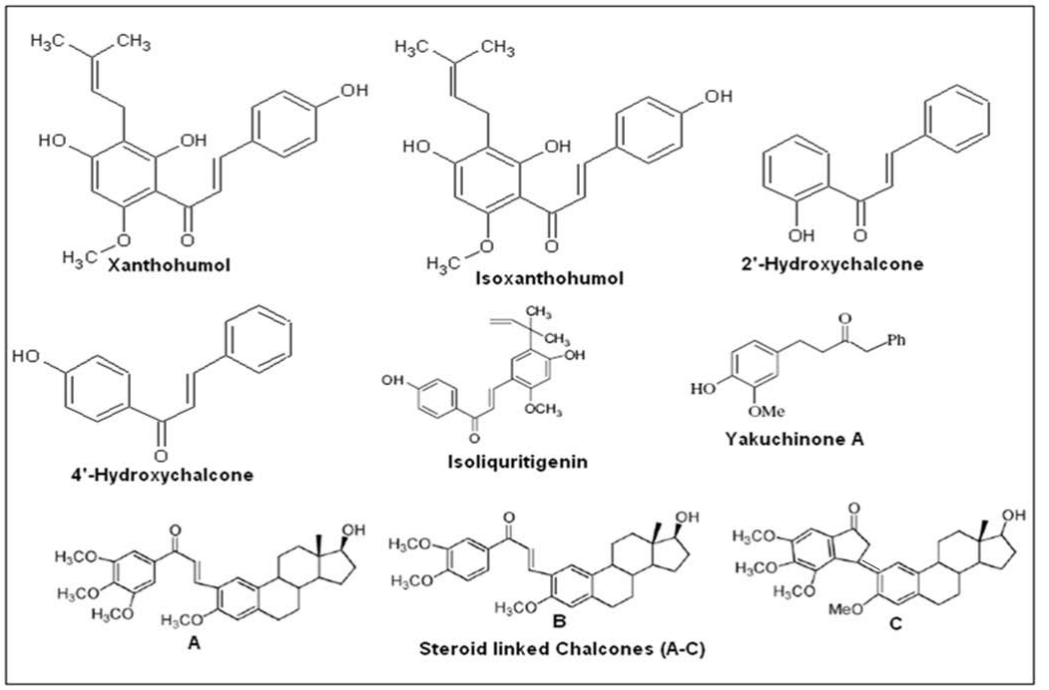
**Structures of some therapeutically active chalcone compounds**.

**Figure 5:**
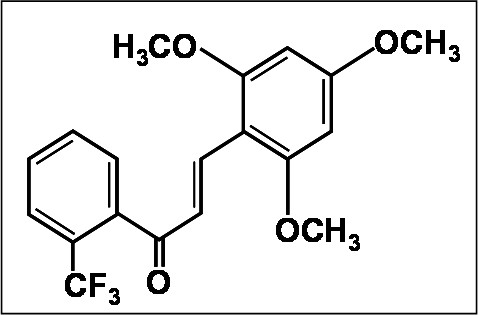
**Fluorinated chalcone: as anti-inflammatory agent**.

Saxena and coworkers grafted chalcone derivatives on estradiol framework some of which showed potent anticancer activity against some human cancer cell lines [87]. Thus, compounds **B **and **C **in Figure 4 show potent activity against estrogen receptor-positive and hormone-dependent human breast cancer cell lines, MCF-7. Chalcone **A **was further modified to yield corresponding indanone derivative **(C) **using the Nazarov reaction, which showed better activity than the parent compound against MCF-7 breast cancer cell line. Active anticancer derivatives were also evaluated for osmotic hemolysis using the erythrocyte as a model system. It was observed that chalcone derivatives showing cytotoxicity against cancer cell lines did not affect the fragility of erythrocytes and hence may be considered as non-toxic to normal cells; however, further research in this area is urgently needed. Nitric oxide production by trimethoxy chalcone derivatives, with various patterns of fluorination, has also been evaluated [88]. One of this compounds, 2, 4, 6-trimethoxy-20-trifluoromethylchalcone, inhibited the production of NO and prostaglandin E2 in lipopolysaccharide-stimulated RAW 264.7 macrophage cells. The inhibition (76.3% inhibition at 10 μM concentration) was dose-dependent without any evidence of a cytotoxic effect. It was suggested that NO reduction was a consequence of inhibition of the expression PGE_2 _accumulation. The fluorinated chalcones tested by Nakamura *et al*. showed 5-lipoxygenase inhibition on rat basophilic leukemia-1 (RBL-1) cells and inhibitory action on Fe^3+^-ADP induced NADPH-dependent lipid peroxidation in rat liver microsomes [89]. The potencies were comparable or better than those of the lead compound, viz. 3,4-dihydroxychalcone. The structure of fluorinated chalcone is presented in Figure 5.

### Why Chalcones are good analogs of Garcinol?

#### 1. Structural Similarity

Chemically, garcinol is a polyisoprenylated chalcone containing two aromatic rings separated by a carbonyl group. The α,β-unsaturated ketone system important for the apoptosis-inducing activity is present between the two rings in case of chalcones but within the ring B in case of garcinol (Figure 2). Garcinol differs from chalcones with the presence of isoprenyl groups, which makes its structure more complex and adds to its antioxidant activity [5].

#### 2. Reaction Similarity

The reducibility of the carbonyl function in chalcones and its relationship to biological activity has been investigated [90]. In quantitative structure-activity relationships (QSAR), the reducibility of the carbonyl function serves as an indirect indicator of the electron density on the carbonyl function. A readily reducible carbonyl group would imply that the carbonyl carbon is electron-deficient. Electron delocalization along the α, β-unsaturated chain would render the β-carbon electron deficient and, accordingly, more susceptible to attack by thiols and other nucleophiles. Thus, one would expect a relationship between the reducibility of the carbonyl bond (for example, measured in terms of reduction peak potentials in cyclic voltametry) and the susceptibility to nucleophilic attack at the β-carbon. On the other hand, *in vivo *reduction of the carbonyl group to an alcohol is unlikely to predominate, as seen from the *in vitro *biotransformation of 4-dimethylamino-4' (imidazol-1-yl) chalcone [91]. In case of garcinol no reactions has been reported but presence of carbonyl group may suggest that such reactions could occur.

#### 3. Similarity in Biological Activities

Chalcones as well as garcinol are reported as potent antioxidants and have been screened for their anti-inflammatory, anti-cancer, anti-HIV, anti-biotic, anti-fungal and anti-tumor activities. Structurally, chalcones are more easily amenable for structural modification and optimization for some selective biological activity than garcinol.

#### 4. Metal Complexation

The synthesis and structural studies of complexes of Co (II), Ni (II), Cu (II), Zn (II) and Cd (II) with substituted chalcones has been reported [92]. In general, for metal complexation reactions, the Schiff derivatives of the chalcones are preferred which not only offer selectivity in metal complexation reactions but also an enhancement in biological activities. As yet no metal complexes of garcinol have been reported.

## Conclusions and perspectives

The chalcone garcinol is a potent antioxidant and anti-cancer agent among its many other biological effects as discussed above. Its structure makes it a very efficient scavenger of oxygen free radicals and an excellent inhibitor of NO. Various biological activities of garcinol have been reported (summarized in Table 1) and most of them relate to its antioxidant nature. More recently, garcinol has generated considerable interest among cancer researchers, and emerging data suggests its ability to protect against chemically-induced carcinogenesis, as well as highlights its potential use as a chemopreventive agent. An interesting observation in this context is its ability to modulate NF-κB, directly or indirectly [29,31]. Since NF-κB is known to be a key player in the progression of human cancers [93,94], its suppression by garcinol indicates a putative potential molecular target of this compound, which requires thorough testing for establishing the scientific rationale for the use of garcinol as an anti-cancer agent prior to its use as a novel therapeutic agent for the treatment of human malignancies. Our preliminary results (unpublished data) suggest an anti-cancer activity of garcinol against human cancer cell lines through induction of apoptosis, and inhibition of NF-κB-DNA binding activity.

**Table 1:** Summary of reported biological activities of garcinol

**Activity**	**Observations**	**Reference**
**Anti-oxidant**	Efficient scavenging of free radicals	Yamaguchi *et al. *[17]
		Yamaguchi *et al.*[28]
		
	Inhibition of NO and H_2_O_2 _production	Sang *et al. *[18]
		
	Inhibition of NO and iNOS Generation	Sang *et al. *[19]
		
	Inhibition of iNOS and COX-2 expression	Liao *et al. *[31]
		
	Inhibition of NO accumulation	Liao *et al. *[30]
		
**Anti-bacterial**	Activity against methicillin-resistant *Staphylococcus aureus*	Iinuma *et al. *[23]
		Rukachaisirikul *et al. *[22]
		
	Efficient killing of *Helicobacter pylori*	Chatterjee *et al. *[20]
		Chatterjee *et al. *[21]
		
**Anti-cancer**	Chemoprevention of colon tumorigenesis	Tanaka *et al. *[34]
		
	Induction of caspase-3-mediated apoptosis	Pan *et al. *[5]
		
	Loss of mitochondrial potential and activation of caspase-3	Matsumoto *et al. *[36]
		
	Induction of apoptosis	Balasubramanyam *et al. *[40]
		
	Inhibition of tongue carcinogenesis	Yoshida *et al. *[38]
		
	Modulation of arachidonic acid metabolism and inhibition of STAT-1	Hong *et al. *[29]
		
	Selective killing of colon cancer cells	Hong *et al. *[33]
		
	Induction of apoptosis and inhibition of cell invasion	Liao *et al. *[35]

Interestingly, induction of apoptosis by garcinol occurs possibly through the activation of caspases as reported [5,36], and our laboratory is beginning to conduct mechanistic studies in support of the role of garcinol as anti-tumor agent against human malignancies, particularly in view of the promising data that has emerged in recent years. Another factor that is starting to generate interest among researchers is the resemblance of the structure of garcinol to that of curcumin (Figure 1). In a direct comparison between these two compounds, it was shown that garcinol has better anti-tumor as well as apoptosis inducing activity [5]. Moreover, garcinol has been shown to modulate various key signaling pathways, as discussed above, which is consistent with the pleiotropic activity of garcinol. In summary, the available literature on garcinol points to its protective role against a number of human ailments and diseases, particularly cancer; however, detailed mechanistic studies are needed in order to fully appreciate the potential beneficial effects of this compound in human health and diseases.

## Declaration of competing interests

The authors declare that they have no competing interests.

## Authors' contributions

SP and FHS conceived of the study and participated in its design. SP, AA and NO conducted the review of literature and drafted the manuscript. Principal investigator, FHS provided the laboratory facility and financial support and helped in the writing and edition of manuscript. All authors read and approved the final manuscript.
